# A systematic review on safety and surgical and anesthetic risks of elective abdominal laparoscopic surgery in infants to guide laparoscopic ovarian tissue harvest for fertility preservation for infants facing gonadotoxic treatment

**DOI:** 10.3389/fonc.2024.1315747

**Published:** 2024-05-28

**Authors:** M. E. Madeleine van der Perk, Anne-Lotte L. F. van der Kooi, Simone L. Broer, Maarten O. Mensink, Annelies M. E. Bos, Marianne D. van de Wetering, Alida F. W. van der Steeg, Marry M. van den Heuvel-Eibrink

**Affiliations:** ^1^ Princess Máxima Center for Pediatric Oncology, Utrecht, Netherlands; ^2^ Department of Obstetrics and Gynecology, Erasmus MC–University Medical Center, Rotterdam, Netherlands; ^3^ Division of Reproductive Endocrinology and Infertility, Department of Obstetrics and Gynecology, University Medical Center (UMC) Utrecht, Utrecht, Netherlands; ^4^ Division of Child Health, Wilhelmina Children’s Hospital, University Medical Center Utrecht, Utrecht, Netherlands

**Keywords:** infants, pediatric oncology, ovarian tissue cryopreservation, fertility preservation, laparoscopy, perioperative complications

## Abstract

**Background:**

Infertility is an important late effect of childhood cancer treatment. Ovarian tissue cryopreservation (OTC) is established as a safe procedure to preserve gonadal tissue in (pre)pubertal girls with cancer at high risk for infertility. However, it is unclear whether elective laparoscopic OTC can also be performed safely in infants <1 year with cancer. This systematic review aims to evaluate the reported risks in infants undergoing elective laparoscopy regarding mortality, and/or critical events (including resuscitation, circulatory, respiratory, neurotoxic, other) during and shortly after surgery.

**Methods:**

This systematic review followed the Preferred reporting Items for Systematic Review and Meta-Analyses (PRISMA) reporting guideline. A systematic literature search in the databases Pubmed and EMbase was performed and updated on February 15^th^, 2023. Search terms included ‘infants’, ‘intubation’, ‘laparoscopy’, ‘mortality’, ‘critical events’, ‘comorbidities’ and their synonyms. Papers published in English since 2000 and describing at least 50 patients under the age of 1 year undergoing laparoscopic surgery were included. Articles were excluded when the majority of patients had congenital abnormalities. Quality of the studies was assessed using the QUIPS risk of bias tool.

**Results:**

The Pubmed and Embase databases yielded a total of 12,401 unique articles, which after screening on title and abstract resulted in 471 articles to be selected for full text screening. Ten articles met the inclusion criteria for this systematic review, which included 1778 infants <1 years undergoing elective laparoscopic surgery. Mortality occurred once (death not surgery-related), resuscitation in none and critical events in 53/1778 of the procedures.

**Conclusion:**

The results from this review illustrate that morbidity and mortality in infants without extensive comorbidities during and just after elective laparoscopic procedures seem limited, indicating that the advantages of performing elective laparoscopic OTC for infants with cancer at high risk of gonadal damage may outweigh the anesthetic and surgical risks of laparoscopic surgery in this age group.

## Introduction

Survival rates of childhood cancer have increased up to 80% (1). However, up to 75% of childhood cancer survivors (CCS) develop one or more late effects such as cardiomyopathy, hypertension, as well as gonadal damage leading to premature ovarian insufficiency and consequently impaired fertility (2, 3). Infertility is rated one of the most important and impairing late effects according to patients, parents of children with cancer and survivors, and is highly associated with decreased quality of life (4, 5). Currently, ovarian tissue cryopreservation (OTC) is often pursued by laparoscopy as an established safe procedure to preserve gonadal tissue in (pre)pubertal girls with cancer at high risk for infertility (6–8). Due to the small size of the ovaries a unilateral oophorectomy or salpingo-oophorectomy is usually performed in prepubertal girls (6, 7, 9, 10). However, currently no international consensus has been published regarding best practice in infants (9, 10). For these girls, less invasive fertility preservation techniques available to adult women, such as oocyte or embryo cryopreservation, are not feasible. Even though no lower age limit for performing OTC is recommended in guidelines (8, 11), and the American Society for Reproductive Medicine reported in 2019 that OTC should no longer be considered experimental, it may be challenging to implement an OTC program due to the perceived risk associated with ovarian tissue harvest in children, typically performed by laparoscopy. Some countries are cautious to perform such an elective procedure in infants under the age of 12 months due to the reported increased risk of perioperative critical events and severe complications after surgery in general in infants based on large pediatric cohort studies (12, 13). As such cohort studies included all types of anesthesia including high risk surgery and all infants, including prematurely born infants or infants with congenital abnormalities, the results may not be representative of elective laparoscopic procedures for OTC in children with cancer (12, 13).

Therefore, this review aimed to evaluate the available evidence of surgical, anesthetic and neurotoxic complication risk in patients undergoing elective laparoscopic surgery under the age of 1 year of only non-high risk surgeries. We aimed to specifically answer the question whether infants undergoing elective laparoscopy are at risk of mortality during and in the first week after surgery, and of critical events (including resuscitation, circulatory, respiratory, neurotoxic, other) during and within the first 24 hours after the surgery. By doing so, we aimed to offer evidence based arguments on the question whether an elective laparoscopic OTC in infants younger than 12 months would be safe.

## Materials and methods

### Literature search strategy

A systematic electronic literature search was performed in December 2019 and updated on February 15^th^, 2023 in the databases Pubmed and EMbase including Medline. Medical Subject Heading (MeSH), Embase subject heading (Emtree) and Title/Abstract (TiAb) terms were applied in the research strategy to detect articles that mention outcome or complications after laparoscopic surgery under general anesthesia in children under the age of 1 year. The following search terms and their synonyms were included: infants (not limited to gender), laparoscopy (not limited to location), intubation, critical events, mortality, comorbidity. The complete search syntaxes are provided in **Supplementary Texts 1**, **2**. Cross reference checks were performed to identify additional potentially relevant articles. This systematic review followed the Preferred Reporting Items for Systematic Review and Meta-Analyses (PRISMA) reporting guideline (14).

### Inclusion and exclusion criteria

We included papers published since 2000, as advances in perioperative safety and developments in pediatric anesthesia over the past 30 years make older studies unrepresentative for the current clinical setting. An example is monitoring by a pulse oximeter, which only became standard care in anesthesia since the late 1990s (15, 16). Articles in English including at least 50 well documented patients under the age of 1 year undergoing elective laparoscopic surgery were included. We decided to exclude articles when the cohort consisted of >75% prematurely born infants, or >50% infants with severe congenital abnormalities, including neurological, cardiac or pulmonary disease, or low- and middle-income countries (LMIC) settings. We excluded thoracoscopic procedures, since they are not our focus of interest as the OTC procedure is performed using abdominal laparoscopy and is hardly ever combined with a thoracoscopic procedure. Additionally, thoracoscopic procedures pose different challenges, e.g. deflation of a lung, compared to laparoscopic abdominal procedures and may therefore not be comparable regarding risks. Details on inclusion and exclusion criteria according to the PICOTS format can be found in **Table 1** (17). Conference abstracts, systematic reviews, book chapters, articles without full text, and case reports were excluded.

**Table 1:** Inclusion and exclusion criteria using the PICOTS criteria (17).

PICOTS	Inclusion	Exclusion
Population	≥75% of the population aged <1 year or subanalyses for subgroup <1 year N=50 < 1 year (of total or subgroup of study)	>75% Prematurely born infants > 50% of population high risk for perioperative events including congenital (major/severe) heart disease, hypoplastic left heart, pulmonary hypertension, severe syndromes including neurocognitive impairment (unless healthy subgroup is separately analyzed)
Intervention	Elective simple abdominal laparoscopic surgery	Open/laparotomic surgery ENT surgery Thoracoscopic surgery Laryngeal surgery Only LMA airways used
Comparator	Not applicable	Not applicable
Outcomes	**Mortality** **Critical events** (CTCAE grade >2) including: - Resuscitation - Hypotension - Hypoxia - Neurotoxicity	Lack of focus on perioperative complications Primary focus on a surgical or anesthetic technique (or comparing techniques) Evaluation of surgical or anesthetic learning curves
Timing	Before the age of 1 year	After the age of 1 year
Setting	Pediatric elective non-high risk surgery setting	Emergency room NICU patients Experience in developing/LMIC country Non-English, Reviews, systematic reviews, narrative reviews, literature reviews, short communications, guidelines, case reports, case series

ENT, ear nose throat; LMA, Laryngeal Mask Airway; NICU, neonatal intensive care unit; LMIC, low middle income country.

### Data extraction

All articles were independently screened on title/abstract and full text and reviewed by at least two authors (M.E.M.v.d.P, A.L.F.v.d.K, A.M.E.B, S.L.B, A.W.F.v.d.S., M.O.M. and M.M.v.d.H.E) using the screening tool Rayyan (18). Disagreements between the reviewers were resolved by discussion and reaching consensus including a third author if necessary, and discussion with the full author group. Abstracts selected for full text screening were selected based on the inclusion criteria in **Table 1**. From the selected papers, the following data were collected: sample size, patient characteristics (age and weight at time of the surgery), diagnosis and type of surgery, airway and anesthesia details, confounding factors including American Society of Anesthesiologists (ASA) score and comorbidities. Complications occurring within the first 24 hours and the first week were recorded. Complications included perioperative mortality in the first week after start of anesthesia. Complications such as resuscitation/cardiac arrest and critical events during and within the first 24 hours were defined as respiratory, circulatory and/or neurological events needing serious intervention and/or with possible negative outcomes or consequences.

### Assessment of study quality

A risk of bias assessment was performed to define the quality of the included publications. We used the QUIPS tool as previously described by Hayden et al. (19). Six different domains were included: study participation, study attrition, prognostic factor measurement, outcome measurement, study confounding, and statistical analysis and reporting (19). The papers were graded for separate domains as having high, moderate or low level of bias. According to the recommendation of Hayden et al., the most relevant domains were defined in advance (study participation, prognostic factor measurement, outcome measurement, and study confounding) to judge the overall risk of bias in the included studies (19).

## Results

### Literature search

**Figure 1:**
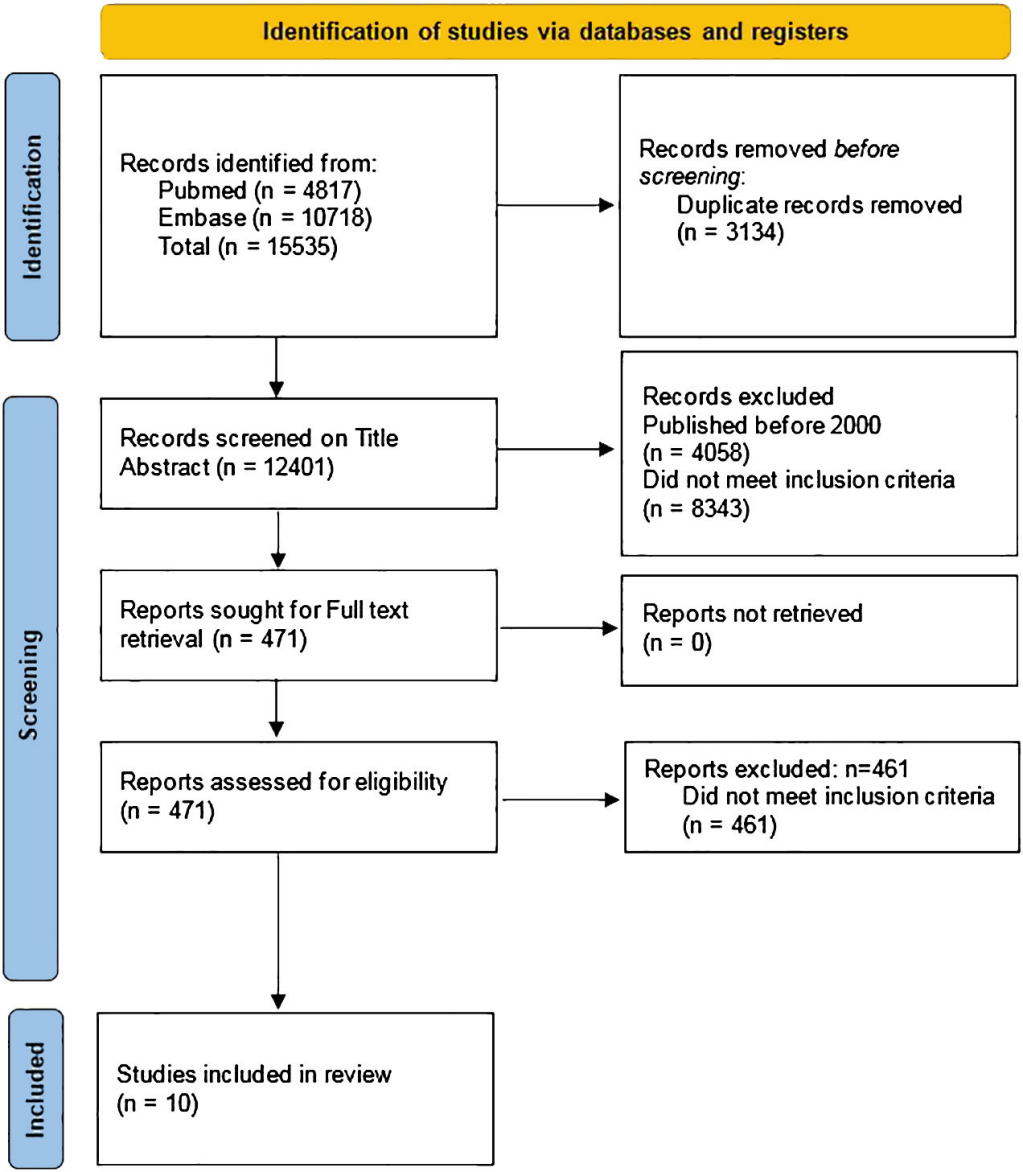
PRISMA 2020 flow diagram of the search strategy (12).

The initial and updated search identified a total of 15,535 articles by February 15^th^, 2023, 4817 from the Pubmed database and 10,718 from the Embase database. After removal of 3134 duplicates, 12,401 articles were screened on title and abstract and 471 articles were subsequently screened on full text. Ten articles fulfilled all inclusion criteria and were included in this systematic review (**Figure 1**) (20–29).

### Characteristics of included studies

The included studies discussed surgical and anesthetic risks of various abdominal laparoscopic surgeries in 1778 children aged <1 years. The results are presented in **Table 2**; **Supplementary Tables 1**–**10** (20–29). The reported laparoscopic surgeries included transanal endorectal pull-through (TERPT) for Hirschsprung disease, pyloric stenosis repair/pyloromyotomy, gastrostomy, congenital megacolon, diaphragmatic hernia, malrotation and inguinal hernia repair. Described methods of anesthesia included general anesthesia only or general anesthesia combined with regional anesthesia. Methods to secure the airway were only reported by 2 studies and both used endotracheal intubation (21, 22). Confounders were inconsistently reported and when reported, results had rarely been corrected for these confounding factors (20, 22, 24, 26–28). Most studies scored a moderate risk of bias (22–26, 28), three a high risk of bias (20, 21, 29) and only one study a low risk of bias (27) (**Table 3**). Insufficiently detailed reporting of prognostic factors, and not reporting confounders were the most common causes for high risk of bias.

**Table 2:** Summary of patient characteristics and risk of bias assessment of included studies.

Authors	Eligible participants ^a^	Age at study ^b^	Weight at study ^b^	Diagnosis, anesthesia method and intervention	Airway	Surgery/anesthesia time	Confounders	Complications anesthesia and surgery		
								Mortality	Resuscitation	Critical events
Beltman (20)	77 (79)	105d † [IQR: 82d]	NR	Transanal endorectal pull-through (TERPT) for Hirschsprung disease	NR	OR time without vs with complications 170 vs 183min [IQR: 74 vs 88]	NR	0	NR	Tachycardia n=1, apnea and stridor n=1/77 = 1.3%
Chou (21)	82	2.25 mo (1d-11.4 mo)	4.2kg (2-11kg)	Various, mainly abdominal	ETT	Surgery time: 3.5h (1-7.5h) Insufflation time: 2.0h (0.2-5.0h)	NR	0 **^d^**	0	0
Disma (22)	63	35 d [IQR: 28-44] †	3.8 kg [IQR: 3.4-4.2] †	Pyloric stenosis repair	ETT n=307 (97.8%)	NR	NR ^m^	0	NR ^h^	NR ^h^.
Fraser (23)	365	All <3mo 5.9 w [IQR: 4.3-8.8]	3.9 kg [IQR: 3.4-4.6]	General for laparoscopic pyloromyotomy (n=246) or inguinal repair (n=119)	NR	OR time: 20 min [IQR: 15-28]	NR	0	NR	N=1 bowel injury (0.2%)(inguinal hernia repair) N=2 hypotension + bradycardia (response to insufflation) resolved w. desufflation (0.5%)(pyloromyotomy) N=0 CO^2^ embolism.
Kalfa (25)	204	16d (0-28d)	3386g (2200-5896)	Anesthesia NR, various surgeries	NR	Mean OR time: 47-60min	NR	0	0	20/204 (9.8%) **^c^** related to pneumo-peritoneum (incl desaturation (max n=8), hypotension (max n=7), hypothermia (max n=4)(in which bradycardia max n=1), hypercapnia (max n=5), metabolic acidosis (max n=2)) n=NR for laparoscopic specific outcomes
Landisch (26)	105	Mean (SD) 96.3w (70.5)	Mean (SD) 4.39 kg (1.28)	Laparoscopic gastrostomy General anesthesia	NR	OR time: mean 130 min (46.4)	Neurologic deficits 21%, pulmonary diagnosis (36%) and cardiac diagnosis (70%) (not further specified)	0	NR	Pneumonia and post-operative respiratory failure in laparoscopic gastrostomy group (7/105 = 6.5% vs 11/105 = 10.5%)^k^
Meng-Meng (27)	63 (Group A: 30 Group B: 33)	A: 48.12d (8.32) B: 51.27d (9.35)	NR	Surgery for congenital megacolon, congenital diaphragmatic hernia, intestinal malrotation. General anesthesia	NR	OR time: A: 151.21 ± 31.18 B: 155.16 ± 29.2	High risk excluded, ASA I, II included	0	NR	**SO^2^ <90%, n (%) 6/63 = 9.5%** A: 2/30 (6.67%) B: 4/33 (12.12%) p=0.59 **Intraoperative blood loss, mL** A: 24.32 ± 9.83 B: 22.42 ± 7.51 p=0.54 **Postoperative hypothermia, n (%) 4/63 = 6.3%** A: 3/30 (10.00%) B: 1/33 (3.03%) p=0.032
Onwubiko (28)	90	5 mo [IQR: 3–11]	5.2 kg [IQR: 4–8.4]	Laparoscopic gastrojejunal (GJ) tube	NR	NR	n=34 (37.8%) cardiac n=29 (32.2%) respiratory diseases (not further specified)	0 ^i^	NR	NR
Ponsky (29)	649	8.54 w (1d-14mo)	<5kg Average* 3.45 kg	Various, anesthesia NR	NR	Variably per surgery (see **Supplementary Table 9**)	NR ^f^	1 (0.15%)^e^	NR	Various events: n=NR Intraoperative 0.9% Total 3%
Walsh (24)	80	10.5 w (-2.5-44)	5.5 kg (2.1-10.8)	Laparoscopic inguinal hernia repair	NR	≤3mo: OR time 93 min (61-125) >3mo: OR time 83 min (47-146)	12/80 (15%) significant cardiac/respiratory comorbidities ^g^ ≤3mo n=8, >3mo n=4	0	NR	No anesthetic complications
**Total summary**	1778	1d-1y	2kg-11kg	Various including pyloromyotomy inguinal hernia repair, gastrostomy/GJ tube, congenital mega colon, diaphragmatic hernia, intestinal malrotation	ETT or NR	OR time 15min-7.5h	Variable: high risk excluded, only ASA I and II Studies included up to 70% cardiac comorbidities.	1/1778 = 0.056%	0 or NR	Total: 57/1778 = 3.2% Cardiac events (including tachycardia (n=1(20)), bradycardia + hypotension (n=2(23)), bradycardia (n=0-1(25)), hypotension (n=1-7(25)): 4-11/1778 = 0.2-0.4%) Pulmonary events (including apnea and stridor (n=1(20)), hypoxia/desaturation (n=2-8(25), n=6(27)), postoperative respiratory failure (n=11(26)), pneumonia (n=7(26)): 27-33/1778 = 1.5-1.9%) Hypothermia: 0-6.3%(n=4(27), n=0-4(25)) Hypercapnia (n=0-5(25)), metabolic acidosis (n=0-2(25))

Values are presented as median (range)[IQR], unless stated otherwise.

ETT, endotracheal tube; IQR, inter quartile range; NR, not reported; N/A, not available; d, days; mo, months; y, year(s); h, hours; min, minutes; OR, operating room; ASA, American Society of Anesthesiologists; n, number of units (participants/events); GJ, gastrojejunal; vs, versus; CO2, carbon dioxide; SD, standard deviation; SO2, oxygen saturation; mL, milliliter; † not laparoscopic group specific. *average not specified as mean/median.

^a^ Laparoscopic (thoracoscopic excluded); **^b^** median (range)[IQR] unless otherwise specified; ^c^ total 20 laparoscopic and 6 thoracoscopic complications including: Desaturation n=8; hypotension n=7; hypercapnia n=5; hypothermia n=4; metabolic acidosis n=2; ^d^ n=2 not procedure related deaths; ^e^ 1 death after Nissen procedure in child with multiple medical problems and severe pulmonary disease, cause of death was severe pulmonary hypertension. ^f^ Comorbidities not reported, but were present as shown by comorbidities stated for the deceased child. ^g^ further specified as: atrial and/or ventricular septal defects, chronic lung disease ^h^ Outcomes not laparoscopic specific: 1/310 cardiac arrest, 7.3% hypoxemia, 25/310 difficult airway, 3/310 pneumonia, 2/310 hypotonia. Other complications (laparoscopic specific): 2/63 redo operation. ^i^ No procedural related mortality, but total 30 day mortality n=4 (4.4%). ^k^ n was not provided in the manuscript but was calculated based on percentages. ^l^ Comorbidities not further specified, ^m^ not reported for laparoscopic group, for total group incl open (n=314 procedures) 15.2% premature, Other congenital abnormalities 43 (13.7) Medical history of: (not further specified) Respiratory n=4 (1.3) Cardiovascular n=8 (2.5) Metabolic n=51 (16.2) Neurological n=1 (0.3) Renal n=3 (1.0) ASA physical status ≥3 n=42 (13.4). ^n^ not laparoscopic specific, for total group incl open: Low birthweight (< 2500 g) n=20 (19%), co-morbidities n=25 (24%), 19/51 genetically tested patients, were diagnosed with genetic mutation/syndrome (39.2%).

**Table 3:** Risk of Bias assessment.

Authors	Study type	Study era	Eligible participants ^a^	Risk of Bias (RoB)						Total RoB
				SB*	AB	MB*	DB*	SC*	SA	
Beltman (20)	Retrospective cohort study	2005-2020	77 (79)	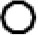	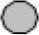	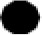	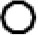	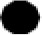	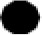	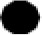
Chou (21)	Retrospective single center	2007-2015	82	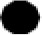	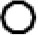	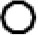	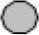	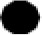	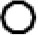	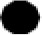
Disma (22)	Prospective observational study	2016-2017	63	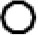	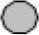	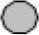	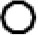	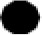	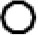	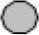
Fraser (23)	Retrospective study	2016-2019	365	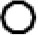	?	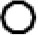	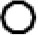	?	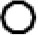	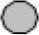
Kalfa (25)	Retrospective cohort study	1993-2005	204	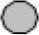	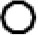	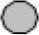	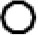	?	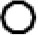	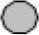
Landisch (26)	Retrospective cohort study	2011-2015	105	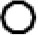	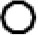	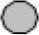	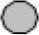	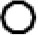	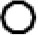	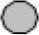
Meng-Meng (27)	Randomized study	2015-2017	63	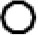	NA	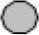	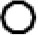	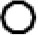	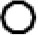	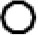
Onwubiko (28)	Retrospective study single institution	2011-2014	90	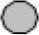	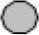	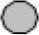	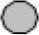	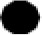	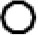	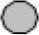
Ponsky (29)	Retrospective database review	1993-2007	649	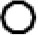	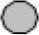	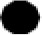	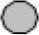	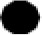	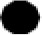	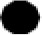
Walsh (24)	Retrospective single surgeon	2013-2018	80	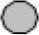	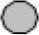	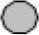	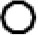	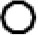	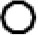	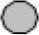

^a^ Laparoscopic (thoracoscopic excluded); SB, selection bias (the study sample adequately represents the population interest); AB, attrition bias (the study data available (i.e.; participants not lost to follow up) adequately represent the study sample); MB, measurement bias (the prognostic factor is measured in a similar way for all participants); DB, detection bias (the outcome of interest is measured in a similar way for all participants); SC, study confounding (important potential confounding factors are appropriately accounted for); SA, statistical analysis (the statistical analysis is appropriate, and all primary outcomes are reported), * in advance, these domains were defined as most relevant domains for our study question. 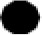
= high risk of bias; 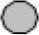
= moderate risk of bias; 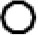
= low risk of bias; ? = unknown/unclear risk of bias. NA, not available.

### Mortality and critical events

#### Mortality

When combining the results of all 10 studies, only 1 death occurred in 1778 (0.056%) reported elective laparoscopic surgeries, which was reported to be not related to the surgery (death due to severe pulmonary hypertension in a child with pre-existent severe pulmonary disease) (**Table 2**; **Supplementary Tables 1**–**10**) (20–29). The deceased infant (death not surgery-related), described in the study of Ponsky et al. (n=649 infants <5kg), had multiple comorbidities and severe pulmonary hypertension after Nissen fundoplication (29). In the other studies no mortality was observed, including the only study with a low risk of bias, a randomized trial including 63 infants with a mean age of approximately 50 days (27).

#### Critical events – overall

Walsh et al. reported no anesthetic complications in a cohort of 80 infants who underwent laparoscopic inguinal hernia repair (24).

#### Critical events – resuscitation

Resuscitation was only specifically reported in two studies (n=82 and n=204) and both studies reported that resuscitation did not occur (21, 25).

#### Critical events – circulatory, respiratory

In total, critical events occurred in 53/1778 (3.2%) patients (20, 23, 25–27). Notably, some patients had a combination of multiple critical events. Since one study reporting 20 adverse events after laparoscopy and 6 after thoracoscopy, did not specify which specific event occurred in which group, for some adverse events ranges are presented (25). Circulatory events consisted of tachycardia (n=1) (20), bradycardia (n=2-3) (23, 25), and hypotension (n=3-9) (25), respiratory events consisted of apnea and stridor (n=1) (20), hypoxia/desaturation (n=8-14) (25, 27), postoperative respiratory failure (n=11) (26) and pneumonia (n=7) (25–27).

#### Critical events – surgical

Surgical events included bowel injury upon entry for inguinal hernia repair (n=1) (23), bladder perforation from trocar insertion (n=NR) and trocar site bleed (n=NR), but also multiple other complications related to the surgery (n=NR) (29), critical events related to the pneumoperitoneum included hypercapnia (n=0-5) (25) and metabolic acidosis (n=0-2) (25). In some series no details are described (25).

#### Critical events – neurological

None of the studies specifically included neurotoxicity after laparoscopic surgery in infants.

#### Critical events – miscellaneous

Other events included hypothermia (n=4-8) (25, 27) (of which 1 led to bradycardia).

Summarizing, mortality was reported in 1 in 1778 (0 to 0.15%) well-described elective non-high risk laparoscopic procedures in infants without congenital abnormalities, and this death was not surgery-related. The range of incidence of perioperative serious events ranged from 0% to 12.12% (desaturation) (**Table 2**).

## Discussion

### Summary of findings

Since no studies have been published comparing the perioperative risk of critical events in children <1 year and >1 year in elective non-high risk laparoscopic surgery, we described the results in this review in the scope of previously published reports on perioperative risks of critical events of elective laparoscopy in children and infants, excluding high risk surgeries. This is challenging as these previously published risks of anesthesia-related mortality (0.1%-3.2%) and severe critical events (2–8%) (12, 30–32) in large cohorts, often did not correct for the invasiveness of the surgery or (congenital or acquired) comorbidities (12, 13, 16, 33–42). Nevertheless, the results from our review illustrate that the incidence of mortality of elective non-high risk laparoscopic procedures in infants without congenital abnormalities is relatively low, 1/1778 cases (0%-0.15%) as is the risk of perioperative events of 3.2% (0% to 12.12% (desaturation)) (**Table 2**). One of the two largest pediatric studies the Anesthesia Practice in Children Observational Trial (APRICOT) study reported a 30 day mortality rate of 0.1%, and perioperative severe critical events rate of 5.2% in children aged 0-15 years during and after diagnostic and surgical procedures, elective, urgent or emergency, under sedation or general anesthesia (results not specified for infants) (12). The NEonate and Children audiT of Anaesthesia pRactice IN Europe (NECTARINE) study included infants under the age of 60 weeks post menstrual age undergoing anesthesia for surgical, non-surgical or diagnostic procedures (including procedures in the ICU) and reported a 90 day mortality rate of 3.2%, and a perioperative serious critical event rate of 35.2%, and 16.3% experienced 1 or more other complications 30 days after the procedure (13). Notably, these two large cohort studies included patients with (congenital) comorbidities, at high risk for severe events, and since they did not report detailed results on complication risk separately for infants without severe (congenital) comorbidities undergoing elective abdominal laparoscopic surgery, the studies could not be included in our review.

### Mortality

The current review shows that the risk of mortality in infants undergoing elective laparoscopy is evidently low (0.056%). The mortality rates in infants <1 year reported for all anesthetic or surgical procedures in other studies varies from 53 to 59.7/10,000 <24h (36, 41, 43, 44) and 5.91-367.4/10,000 after 30 days (36, 37, 41, 43, 45–48). Again, here we excluded high risk surgeries and patients, since in some studies patients <1 year with an ASA score of 3-5 or extensive comorbidities showed highest incidence of perioperative problems and mortality (12, 34–41, 43, 49), while other reports show that surgical procedures can be safely performed in very young children (12, 44). For this subgroup no comparative studies have been published and due to the heterogeneity of the studies no meta-analysis could be performed. Future large scale international studies based on a prospective registry would be of value to evaluate mortality in infants after elective non-high risk laparoscopy.

Remarkably, even in infants (n=45-6325) with comorbidities, the risk of mortality after laparoscopy is low (0-0.6%) (61-70% comorbidities (cardiac risk factors, neurological impairment, hypoplastic left heart (HLHS), prematurity, infants <3-5kg)) (50–57). When including only open or both laparoscopic and open surgery, mortality rates range from 0 in small cohorts (58, 59) to 0.4-4.4% in larger studies (n=151-2967) even in cohorts with many patients with ASA≥3 and minor to severe cardiac risk factors (60–62). Thus, it may be concluded that the risk of surgery-related mortality in infants after laparoscopy is low, even in infants with comorbidities placing them at increased risk for adverse events (53, 60, 63–66). Nonetheless, mortality may occur due to the underlying disease (52).

### Critical events

#### Resuscitation

Resuscitation or cardiac arrest after laparoscopic surgery in infants was specifically reported by two of our included studies, which reassuringly reported 0 resuscitations in 286 patients (21, 25). The event of resuscitation was not reported in the remaining 1492 infants in the other 8 studies (20, 22–24, 26, 27, 29). In the latter studies, it is possible that cardiac arrests did or did not occur but were not structurally reported, or did not occur due to the small size of this sample. Perioperative cardiac arrest related to anesthesia in infants has been reported to occur in 38.6/10,000, ranges being reported from 8.7 to 87.1/10,000 in a recent systematic review (n= 122,196) (44). But again, these cohorts differ from the included population of interest (34, 36, 37, 43, 58, 67). In cohorts including >40% infants <1 year with cardiac risk factors cardiac arrests occurred in 0.9% (n=2967) when undergoing major abdominal and thoracic surgery (60). This may indicate that the risk of resuscitation is reassuringly low in infants undergoing elective non-high risk laparoscopic surgery.

#### Morbidity: Other critical events

Critical events occurred in 53/1778 (3.2%) patients in the included studies, which seems to be lower than reported in other studies describing also open surgeries and anesthetic procedures in infants, in which the rates vary between 4.6% and 30.8% (34, 35, 44–46, 48, 68, 69). The APRICOT study reported a higher rate of cardiovascular and respiratory critical events in neonates (0–1 month) and infants (1 month to 1 year) (12). In neonates cardiovascular complications occurred in 12.1% (12). The NECTARINE study reported perioperative serious critical event in 35.2% of cases and 16.3% experienced 1 or more other complications 30 days after the procedure (13). Notably, endpoint definitions were not consistent between the included studies, which is in line with other literature and critical events included respiratory, cardiovascular and neurological events such as hypoxemia, hypotension, hypo- or hyperthermia, anaphylaxis, intubation problems, vomiting, coma/seizure (34, 35, 45, 46, 48, 68, 69). Some smaller studies (n<50) report a severe perioperative event rate of 0 (51, 59) or 1 (severe hypercapnia) (58) and that laparoscopic surgery or minimal access surgery is safe even in very small infants (56, 57). Currently published risk factors seem to be related to cardiovascular and respiratory complications, which is in line with infant anatomy and physiology (49, 70–72), and in contrast to the risk factors associated with anesthetic use (halothane) or anesthetic procedures in the past (42, 44). None of the included studies reported laryngospasm, despite the fact that in general infants <1 year have an increased risk of laryngospasm (2.7%) compared to older children (35). In general, in children laryngospasms are among the most common critical events occurring during/after general anesthesia (73–75). So overall, also regarding critical events, laparoscopic surgery appears safe for infants.

#### Neurotoxicity

Notably, none of the included studies reported neurotoxicity after laparoscopy, despite the fact that neurotoxicity after anesthesia has received attention in the past (33, 76–83). In 2012 a consensus was published to state that necessary procedures or surgeries in infants and children of preschool age should not be postponed due to fear of neurotoxicity (84, 85). This was supported by the encouraging studies on the long term impact on infants of anesthesia (<1 hour) during inguinal hernia repair (86, 87). The large ongoing international randomized controlled trial (General Anesthesia compared to Spinal anesthesia (GAS) study) and the ongoing Pediatric Anesthesia NeuroDevelopment Assessment (PANDA) study showed that psychomotor development (at age 5 years) was unremarkable (86, 87).

### Strengths and limitations

This is the first systematic review evaluating the surgical and anesthetic risks of elective non-high risk laparoscopic surgery in infants <12 months. It seems that laparoscopic surgery in infants is safe, yet no adequate registry or study has been published to specifically evaluate this. For this review, confounders were not always sufficiently described (26, 28), leading to higher risk of bias in some studies and not all studies systematically reported neurotoxicity, ASA status or resuscitations (12, 34–38, 49, 88–93). Available systematic reviews on critical events and complications in pediatric anesthesia in general revealed already that there is a great variability within and between studies regarding definitions of events and diagnostic criteria for complications (44, 94). This variability between studies may limit generalizability to the pediatric oncology population. The large differences between the studies prohibited a clinically relevant meta-analysis.

#### Considerations specifically for infants with pediatric cancer

The currently available recommendations for fertility preservation in children with cancer do not include any recommendation regarding a safe lower age limit for laparoscopic OTC (8, 11) and anesthetic and surgical risk of laparoscopic OTC specifically in infants <1 year have not been published (95–97). The fact that our review only identified one deceased case (among n=1778 well documented cases) and that only low percentages of circulatory and respiratory events were observed, suggests that elective laparoscopic surgeries can safely be performed in infants <12 months. The available evidence on the risk of anesthesia and abdominal laparoscopic surgery <1 year may suggest that these risks do not outweigh the advantages of performing fertility preservation for those at high risk of gonadal damage and premature ovarian failure. However, since none of the studies studied OTC specifically, it may be argued that the included procedures such as inguinal hernia repair in infants may not be comparable to OTC regarding surgical complexity and complication risk, as children are also suffering from childhood cancer.

Specifically, some infants may not tolerate the pneumoperitoneum (51, 52, 56, 98), and other challenges include the presence of a large abdominal tumor, limiting the operative space and/or impairing pulmonary capacity, or disrupted blood counts in patients with leukemia or after intensive chemotherapy. On the other hand, the offer of OTC may not be withheld from girls with a clear high risk of infertility, such as those needing allogenic stem cell transplantation as primary salvage treatment for juvenile myelomonocytic leukemia (JMML) for instance or whole abdominal radiotherapy for a massive rupture of a nephroblastoma at presentation. Obviously, these risks need to be weighed against the benefit of OTC, especially now that the first live births have been reported after autotransplanation of ovarian tissue, harvested in children (6, 99–103) and the first results of auto-transplantation in prepubertally harvested ovarian tissue look promising (6, 104, 105).

Specific risks in the highly selected patients who are eligible for OTC need to be discussed during counseling for OTC. In addition, risks may be reduced by careful selection of the patients, pursuing these procedures in expert pediatric oncology centers, where an oncofertility team, including pediatric surgeons, gynecologists and pediatric anesthesiologists, estimates the risk and anticipate the best controlled setting. This can include postponing OTC until after the first rounds of chemotherapy, in order to reduce the risk of complications including organ damage or tumor spill, but also to reduce circulating tumor cells in the ovarian tissue in leukemia patients. Furthermore, clear criteria should be in place to ensure laparoscopic OTC can be performed safely, including but not limited to platelet parameters to decrease bleeding risk. Hence, a multidisciplinary and personalized approach and a controlled oncological-surgery-fertility approach is important for all infants (and older girls) in which laparoscopic OTC is considered (60, 106).

## Data availability statement

The original contributions presented in the manuscript are included in the article/**Supplementary Material**. Further inquiries can be directed to the corresponding author.

## Author contributions

MvdP: Conceptualization, Data curation, Formal analysis, Investigation, Methodology, Project administration, Visualization, Writing – original draft, Writing – review & editing. AvdK: Conceptualization, Investigation, Methodology, Supervision, Writing – review & editing. SB: Investigation, Methodology, Writing – review & editing. MM: Investigation, Methodology, Writing – review & editing. AB: Conceptualization, Investigation, Methodology, Supervision, Writing – review & editing. MvdW: Investigation, Writing – review & editing. AvdS: Investigation, Methodology, Writing – review & editing. MvdHE: Conceptualization, Formal analysis, Funding acquisition, Investigation, Methodology, Project administration, Resources, Supervision, Validation, Writing – review & editing.
