# Supplementary material for: A systematic review on safety and surgical and anesthetic risks of elective abdominal laparoscopic surgery in infants to guide laparoscopic ovarian tissue harvest for fertility preservation for infants facing gonadotoxic treatment
Source: Front Oncol. 2024 May 28;14:1315747. doi: 10.3389/fonc.2024.1315747 (PMC11165185; doi:10.3389/fonc.2024.1315747)
Supplement: Supplementary file 1 [file DataSheet_1.docx]

**Supplemental material**

Supplemental text 1: Search syntax Pubmed

Supplemental text 2: Search syntax Embase

Supplemental Table 1: Beltman

Supplemental Table 2: Chou

Supplemental Table 3: Disma

Supplemental Table 4: Fraser

Supplemental Table 5: Kalfa

Supplemental Table 6: Landisch

Supplemental Table 7: Meng-meng

Supplemental Table 8: Onwubiko

Supplemental Table 9: Ponsky

Supplemental Table 10: Walsh

Reference list

**Supplemental text 1. Search syntax Pubmed**

(((((child[MeSH Terms] OR child[Title/Abstract] OR children[Title/Abstract] OR toddler[Title/Abstract] OR toddlers[Title/Abstract] OR baby[Title/Abstract] OR babies[Title/Abstract] OR infant[MeSH Terms] OR infant[Title/Abstract] OR infants[Title/Abstract] OR neonate[Title/Abstract] OR neonates[Title/Abstract] OR neonatal[Title/Abstract] OR premature* [Title/Abstract] OR prematurity[Title/Abstract] OR ELGA[Title/Abstract] OR preterm[Title/Abstract] OR "extremely low gestational age"[Title/Abstract] OR newborn[Title/Abstract] OR newborns[Title/Abstract] OR pediatric[Title/Abstract] OR pediatrics[Title/Abstract] OR paediatric[Title/Abstract] OR paediatrics[Title/Abstract]) AND (intubation[MeSH Terms] OR intubation[Title/Abstract] OR intubate[Title/Abstract] OR intubated[Title/Abstract] OR intubations[Title/Abstract] OR laparoscopy[MeSH Terms] OR laparoscopy[Title/Abstract] OR laparoscopic[Title/Abstract] OR laparoscopies[Title/Abstract] OR laparoscopically[Title/Abstract] OR laparoscopical[Title/Abstract] OR laparoscope[Title/Abstract] OR laparoscopes[Title/Abstract]) AND ("critical event"[Title/Abstract] OR "critical events"[Title/Abstract] OR "adverse event"[Title/Abstract] OR "adverse events"[Title/Abstract] OR "adverse effect"[Title/Abstract] OR "adverse effects"[Title/Abstract] OR mortality[Title/Abstract] OR mortalities[Title/Abstract] OR fatal[Title/Abstract] OR fatality[Title/Abstract] OR fatalities[Title/Abstract] OR death[Title/Abstract] OR deaths[Title/Abstract] OR death[MeSH Terms] OR mortality[MeSH Terms] OR "long term adverse effects"[MeSH Terms] OR neurotoxic[Title/Abstract] OR neurotoxicity[Title/Abstract] OR neurotoxicities[Title/Abstract] OR dead[Title/Abstract])))) AND ((risk[Title/Abstract] OR risks[Title/Abstract] OR incidence[Title/Abstract] OR incidences[Title/Abstract] OR complication[Title/Abstract] OR complications[Title/Abstract] OR comorbidity[Title/Abstract] OR comorbidities[Title/Abstract] OR outcome[Title/Abstract] OR outcomes[Title/Abstract] OR "Risk"[Mesh] OR "Incidence"[Mesh] OR "Comorbidity"[Mesh])))

**Supplemental text 2. Search syntax EMBASE**

(child:ti,ab,kw OR children:ti,ab,kw OR toddler:ti,ab,kw OR toddlers:ti,ab,kw OR infant:ti,ab,kw OR infants:ti,ab,kw OR neonate:ti,ab,kw OR neonates:ti,ab,kw OR neonatal:ti,ab,kw OR baby:ti,ab,kw OR babies:ti,ab,kw OR premature:ti,ab,kw OR prematurity:ti,ab,kw OR elga:ti,ab,kw OR preterm:ti,ab,kw OR 'extremely low gestational age':ti,ab,kw OR newborn:ti,ab,kw OR newborns:ti,ab,kw OR pediatric:ti,ab,kw OR pediatrics:ti,ab,kw OR paediatric:ti,ab,kw OR paediatrics:ti,ab,kw OR preterms:ti,ab,kw OR prematurely:ti,ab,kw OR prematures:ti,ab,kw OR premature*:ti,ab,kw OR 'child'/exp OR 'infant'/exp OR 'newborn'/exp OR 'baby'/exp OR 'toddler'/exp OR 'prematurity'/exp OR 'extremely low gestational age'/exp OR 'pediatrics'/exp) AND (intubation:ti,ab,kw OR 'respiratory tract intubation':ti,ab,kw OR intubate:ti,ab,kw OR intubated:ti,ab,kw OR intubations:ti,ab,kw OR laparoscopy:ti,ab,kw OR 'laparoscopic surgery':ti,ab,kw OR laparoscopic:ti,ab,kw OR laparoscopies:ti,ab,kw OR laparoscopically:ti,ab,kw OR laparoscopical:ti,ab,kw OR laparoscope:ti,ab,kw OR laparoscopes:ti,ab,kw OR 'intubation'/exp OR 'respiratory tract intubation'/exp OR 'endotracheal intubation'/exp OR 'laparoscopy'/exp OR 'laparoscopic surgery'/exp OR 'laparoscope'/exp) AND ('critical event':ti,ab,kw OR 'critical events':ti,ab,kw OR 'adverse event':ti,ab,kw OR 'adverse events':ti,ab,kw OR 'adverse effect':ti,ab,kw OR 'adverse effects':ti,ab,kw OR mortality:ti,ab,kw OR mortalities:ti,ab,kw OR fatal:ti,ab,kw OR fatality:ti,ab,kw OR fatalities:ti,ab,kw OR death:ti,ab,kw OR deaths:ti,ab,kw OR 'long term adverse effect':ti,ab,kw OR 'long term adverse effects':ti,ab,kw OR neurotoxic:ti,ab,kw OR neurotoxicity:ti,ab,kw OR neurotoxicities:ti,ab,kw OR dead:ti,ab,kw OR 'adverse event'/exp OR 'mortality'/exp OR 'fatality'/exp OR 'death'/exp OR 'neurotoxicity'/exp) AND (risk:ti,ab,kw OR risks:ti,ab,kw OR incidence:ti,ab,kw OR incidences:ti,ab,kw OR complication:ti,ab,kw OR complications:ti,ab,kw OR comorbidity:ti,ab,kw OR comorbidities:ti,ab,kw OR outcome:ti,ab,kw OR outcomes:ti,ab,kw OR 'risk'/exp OR 'incidence'/exp OR 'complication'/exp OR 'comorbidity'/exp)

Suppl Table 1

| **Are infants at increased risk of**  **critical events during elective intubation or laparoscopy?** | | | | | |
| --- | --- | --- | --- | --- | --- |
| **Beltman, L. *et al.*** Risk factors for short-term complications graded by Clavien-Dindo after transanal endorectal pull-through in patients with Hirschsprung disease*. J Pediatr Surg* ***57****, 1460-1466 (2022)*  ^1^ | | | | | |
| **Study design**  **Treatment era**  **Years of follow-up** | | **Participants** **laparoscopy and intubation** | | **Treatment** | |
| Study design:  Retrospective cohort study Netherlands  Study era:  15 year period (November 2005-June 2020)  Follow-up:  Minimum follow up of 30 days after corrective surgery  Other information †:  22/106 (21%) developed 35 complications, including 2 patients (1.8%) that deceased (both laparotomy). postoperative rectal irrigation leading to perforation is suspected cause of death in both patients.  6 (6%) had a minor (Clavien-Dindo (CD) < 3) and 16 (15%) a major (CD ≥3) complication. Anastomotic leakage n = 4, (11%), abdominal abscess n = 3, (9%) anastomotic stricture n = 3, (9%) occurred most frequently.  Predictive factors for complication: older age at surgery (OR 1.03 1.00–1.01, p = 0.041), laparotomy-assisted surgery (OR 12.65, CI 1.712–93.07, p = 0.013) and long-segment HD (OR 4.09 CI 1.09–15.39, p = 0.037). | | Type and number of non-participants:  66 received other surgical correction for HD, 4 had surgery performed elsewhere.  Type and number of participants:  All consecutive patients surgically treated with TERPT in the AMC and VUMC Amsterdam.  N=106, laparoscopic: n= 79 (77 in abstract)  Diagnoses/type intervention:  Hirschsprung disease  Age at intervention:  105 days (IQR: 82 days) (n=106)†  Male n=80 (75%)†  Weight at surgery: NR  Controls:  17 transanal only  10 (12 in abstract/full text) open/laparotomy assisted  Additional study characteristics/ confounders:  Inclusion: all consecutive patients treated surgically with TERPT for HD in 1 of 2 hospitals with minimum follow-up time of 30 days (or died before this date)  Exclusion criteria: operated with different approach (not TERPT), surgery in another hospital, when no surgical report was available, no histopathological confirmation of HD, and patients that did not provide informed consent.  Confounders: †  Mean gestational age: 39 weeks (range 32–42 weeks)  Mean birth weight: 3314 g (SD = 618 g).  Low birthweight ( < 2500 g) n=20 (19%)  Co-morbidities n=25 (24%), 19/51 patients genetically tested, diagnosed with genetic mutation/syndrome (39.2%). Syndrome n=10, Down ´s syndrome n=8, Mowat-Wilson n=1, deletion of chromosome 22q11 n=1.  Median age at diagnosis = 22 days (IQR: 37 days)  Preoperative enterocolitis n=8 (7.5%). | | Surgery:  Transanal endorectal pull-through (TERPT) for Hirschsprung disease (HD)  17 transanal only, 79 (77 in abstract/full text vs table 1) laparoscopic assisted 10 (12 in abstract/full text) laparotomy assisted, data for 77 laparoscopic only.  91 pt received antibiotics perioperatively  Without complications operative time: 170min (IQR: 74), total time: 239 min (IQR: 71); with complications operative time: 183min (IQR: 88), total 238min (IQR: 115)†  Airway management:  NR  Anesthesia:  NR  Other:  Surgeon decided laparotomy or laparoscopy assistance. | |
| **Complications mortality (1w), resuscitation (24h), critical events (24h)** | | | | | |
| **Main outcomes**  **Intubation** | **Main outcomes**  **Laparoscopy** | | **Additional remarks** | |  |
| **NR**  Outcome definitions:  -  Results:  -  Risk factors/ determinants:  - | Outcome definitions:  Complications emerging <30 days (Clavien-Dindo (CD))  Results:  **Mortality:**  Laparoscopic 0/79 (0/77)  Open: 2/10 (2/12)  **Resuscitation** (24h): NR  **Critical events** (24h*):  Table 1 specifies: Complications in 14/79 laparoscopic procedures. No complications in 65/79.  Complications registered for all (not categorized per surgical approach)  1: Upper resp tract infection CD 2  1: ileus + sepsis CD 3 and 4.  1: abdominal compartment syndrome + multi organ failure CD 5 and 5.  1: perforation and sepsis CD 4 and 5  1: Persistent tachycardia + discomfort despite analgesia requiring reoperation; no cause found.  1: apnea + stridor - Transfer to PICU for respiratory monitoring  * = timing not available  Risk factors/ determinants:  - | | Strengths:  All consecutive patients included.  Dutch hospitals, generalizable to population of interest.  Details of patients who died described.  Limitations:  Perioperative complications not adequately described, only laparoscopic specific information available for mortality.  Timing of complications not specified.  Complications not subspecified for laparoscopic group.  **Risk of bias**  A. Selection bias:  low risk  Reason: All consecutive patients included. Exclusion criteria well specified.  B. Attrition bias:  moderate risk  Reason: excluded patients not adequately described, only reason for exclusion reported.    C. Measurement bias:  high risk  Reason: Airway and anesthesia methods not described. Laparoscopic technique not described. | | D. Detection bias:  low risk  Reason: complications graded by the CD criteria.  E. Confounding:  high risk  Reason: confounders were described, but not in relation to the complications which occurred. Only the deceased patients were described in more detail.  F. Statistical analysis:  high risk  Reason: discrepancies between numbers in the abstract and full text and table 1 in the full text. In full text number of laparoscopic procedures n=79 and open assisted n=10. In the abstract number of laparoscopic procedures n=77 and open assisted n=12. |

† not laparoscopic data specifically.

Suppl. Table 2

| **Are infants at increased risk of**  **critical events during elective intubation or laparoscopy?** | | | | | | |
| --- | --- | --- | --- | --- | --- | --- |
| **Chou, C. M., Yeh, C. M., Huang, S. Y. & Chen, H. C.** Perioperative parameter analysis of neonates and infants receiving laparoscopic surgery. *J Chin Med Assoc* **79**, 559-564 (2016). ^2^ | | | | | | |
| **Study design**  **Treatment era**  **Years of follow-up** | | **Participants** **laparoscopy and intubation** | | **Treatment** | |  |
| Study design:  Retrospective single center Taiwan  Study era:  Jan 2007 – August 2015  Follow-up:  Median 23.4 months (1mo-102.6mo)  Other information:  Other complications  Immediate operative complications n=5.  1 colorectal anastomotic leakage (Hirschsprung disease) --> diversion ileostomy  1 Y-enteroenterostomy leakage (biliary atresia)  --> exploratory laparotomy for anastomosis revision.  2 Urine leakage (ureteropelvic junction stenosis) --> 1 local drainage, 1 percutaneous nephrostomy  1 anorectal malformation urine retention recovered gradually. | | Type and number of non-participants:  NR  Type and number of participants:  Neonates and infants <1 year N=82  42 male, 40 female  Diagnoses/type intervention:  CHD abd laparoscopy etc various  Age at intervention:  Median age 2.2 mo, all <1y (range: 1d-11.4mo)  Median weight: 4.2kg (range: 2-11kg)  Controls:  NA  Additional study characteristics/ confounders:  NR: Prematures appear to have been excluded (see weight) | | Surgery:  Various, primarily abdominal: supine or lithotomy position. Transumbilical port introduced using open Hasson technique. A 5-mm scope in transumbilical port, other two or three work ports (5 mm or 3 mm) according to operational needs. Pneumoperitoneal space using CO2 insufflation, pressure 8-10 mmHg in neonates, 10-12 mmHg in infants.  Partial adrenalectomy: 1; Anorectal malformation: Laparoscopic-assisted pull-through 3; Biliary atresia: Kasai operation 6, Excision of choledochal cyst and roux-en-y hepaticojejunostomy 9, Duodenoduodenostomy 1; Congenital diaphragmatic hernia: Primary repair 1, Patch repair 1, Plication of diaphragm 2, Nissen fundoplication and gastrostomy 1, Gastrostomy 2; Hirschsprung disease: Swenson procedure 10; Hiatal hernia: Primary repair and Toupet fundoplication 5; Pyloromyotomy: 5; Segmental resection and anastomosis of ileal atresia: 3; Excision of intra-abdominal lymphangioma tumor: 1; Surgical Intussusception reduction: 5; Unroofing liver cyst: 2; Intraoperative cholangiogram: 1; Partial nephrectomy: 5; Ovarian cyst/teratoma: Partial/total oophorectomy 4, Laparoscopic-assisted orchiopexy 6; Ureteropelvic junction stenosis: Dismembered pyeloplasty 7; Vesicoureteral reflux: Ureteral reimplantation 1.  Airway management:  Endotracheal tube with capnometer  Anesthesia:  Unclear  Other:  Median operative time: 3.5h (1-7.5h)  Median insufflation time: 2.0h (0.2-5.0h)  Mean intraoperative end-tidal carbon dioxide (EtCO2) level: 37.6 mmHg  Median body temp: 35.8C; mean PIP: 23.3cmH2O. | |  |
| **Complications mortality (1w), resuscitation (24h), critical events (24h)** | | | | | | |
| **Main outcomes**  **Intubation** | **Main outcomes**  **Laparoscopy** | | **Additional remarks** | |  | |
| **NR**  Outcome definitions:  -  Results:  -  Risk factors/ determinants:  - | Outcome definitions:  Perioperative complications  **Mortality**  Results:  n=0 Mortality from laparoscopy  Other mortality: n=2  1 died from severe sepsis caused by central venous catheter infection 1 month after laparoscopic hiatal hernia repair.  1 died from congenital heart disease 1 year after laparoscopic Hirschsprung surgery  **Resuscitation**  Results:  n=0  **Critical events**  Results:  Hypotension n=0  Hypercarbia-related intraoperative respiratory acidosis (n=3) and transient hypothermia (BT<35C) (n=4). All recovered soon after insufflation was stopped or pressure decreased by 2mmHg.  Risk factors/ determinants:  Blood loss was minimal in most patients, except 1 partial nephrectomy: estimated blood loss 40 mL --> blood transfusion. | | Strengths:  Abdominal laparoscopic surgery in infants  Limitations:  Unclear selection criteria  **Risk of bias**  A. Selection bias:  high risk  Reason: no exclusion criteria mentioned, we presume all patients in time period were included. Since various laparoscopic procedures were included.  B. Attrition bias:  low risk  Reason: Follow-up is a adequately described for our outcome, reason to loss to FU is not reported, but is a retrospective study.    C. Measurement bias:  low risk  Reason: method of laparoscopy adequately described, airway very sparsely described, but the capnometer was placed at the ET tube. Not reported if this data is available for all. | | D. Detection bias:  moderate risk  Reason: outcome definitions were not described in detail.  E. Confounding:  high risk  Reason: comorbidities not described. Only 1 death in child with heart disease.  F. Statistical analysis:  low risk  Reason: retrospective analyses and descriptive statistics adequately reported. | |

Suppl. Table 3

| **Are infants at increased risk of**  **critical events during elective intubation or laparoscopy?** | | | | | |  |
| --- | --- | --- | --- | --- | --- | --- |
| **Disma, N. *et al.*** Neonates undergoing pyloric stenosis repair are at increased risk of difficult airway management: secondary analysis of the NEonate and Children audiT of Anaesthesia pRactice IN Europe. *Br J Anaesth* **129**, 734-739 (2022). ^3^ | | | | | |  |
| **Study design**  **Treatment era**  **Years of follow-up** | | **Participants** **laparoscopy** | | **Treatment** | |  |
| Study design:  Prospective observational study  NEonate and Children audiT of Anesthesia pRactice In Europe (NECTARINE) database: total: 165 participating centres from 31 European countries. Each participating center included patients for 3 months.  Pyloric stenosis: 110 centers in 29 countries: 61% of centers performed <3 procedures during 3 months.  Study era:  March 2016 and January 2017  Follow-up:  Up to 90 days:  N=292 30 days, n= 223 90 days.  Other information:  Inclusion: For complete NECTARINE study, all neonates and infants <60 weeks post-menstrual age (gestational age at birth + chronological age) undergoing anaesthesia for surgical and diagnostic (non-surgical) procedures, in the operating room (OR), ICU, or diagnostic suite (ref 7) | | Type and number of non-participants:  NR  Type and number of participants:  N=310 patients total primary pyloric stenosis repair n=314 procedures, lap: n=63  Diagnoses/type intervention:  Hypertrophic pyloric stenosis repair  Age at intervention:  35 (IQR: 28-44) days  Weight 3.8 (IQR: 3.4-4.2) kg  Male n=253 (80.6%)  Controls:  N=5299 non pyloric stenosis surgery  Additional study characteristics/ confounders: not laparoscopic specific  Confounders for complete group: n=314  ASA physical status 1 or 2 n=272 (87%)  15.2% born prematurely  Other congenital abnormalities 43 (13.7%)  Medical history of: (not further specified)  Respiratory n=4 (1.3%)  Cardiovascular n=8 (2.5%)  Metabolic n=51 (16.2%)  Neurological n=1 (0.3%)  Renal n=3 (1.0%)  ASA physical status ≥3 n=42 (13.4%) | | Surgery:  Pyloric stenosis repair  Airway management:  Large variability in neuromuscular blocking agents for tracheal intubation (n=51 intubation no neuromuscular blocking agents).  Tracheal intubation n=307 (97.8%) (165 cuffed, 142 uncuffed; oral: 290, nasal: 17)  Supraglottic airway: 2 (0.6%)  Face mask: 3 (1.0%)  Nasal probe/CPAP/NIV: 1 (0.3%)  None: 1 (0.3%)  Anesthesia:  General anesthesia (alone or combined with regional): 312 procedures. N=2 regional alone.  Study did not specifically investigate mode of anaesthesia induction: 63.5%: intravenous induction, 36.5% inhalation induction  Other:  - | |  |
| **Complications mortality (1w), resuscitation (24h), critical events (24h)** | | | | | |  |
| **Main outcomes**  **Intubation** | **Main outcomes**  **Laparoscopy** | | **Additional remarks** | |  | |
| **NR**  Outcome definitions:  -  Results:  -  Risk factors/ determinants:  - | Outcome definitions:  Hypoxaemia: decrease in oxygenation saturation <90%  Severe hypoxaemia<85%. Thirty-day morbidity:  New onset neurological, respiratory, cardiovascular, renal, hepatic, and surgical complications and any readmission to ICU. Mortality at 30 and 90 days.  Results:  **Mortality** n=0  **Resuscitation**/cardiac arrest laparoscopic: NR  2/63 (3.4%) surgical complication (redo surgery)(8 (3.6%) open)  *Not laparoscopic specific:*  Mortality n=0  Complications 30-day: 5.6% (95% CI, 3.2-8.9%): n=2 neurological complication (new onset hypotonia), n=3 respiratory complication (pneumonia), n=1 cardiac arrest.  Difficult airway management n=25 (7.96%, [95% CI, 5.2-11.5%]), sign higher than other surgeries (4.41%) [95% CI, 3.85-5.02]; RR=1.81 [95% CI, 1.21-2.69]; P=0.004).  Hypoxemia at induction comparable between this cohort and other surgeries. overall incidence: 7.3% (95% CI, 4.7-10.8), with moderate (n=8, SpO2 <85%) or severe hypoxemia (n=2, SpO2 <80%) in 34.8%.  Risk factors/ determinants:  Pyloric stenosis repairs have increased risk of difficult airway. | | Strengths:  Large European study  Limitations:  Most outcome not specified for laparoscopic procedures.  Not corrected for confounders.  **Risk of bias**  A. Selection bias:  low risk  Reason: Large cohort, prospective study, all patients included. While complete cohort also includes non-surgical, this subcohort contains only surgical.  B. Attrition bias:  moderate risk  Reason: primary outcomes were available for all children, follow-up 30 days n=292, 90 days n=223. No data on who did not complete follow-up, less important for our outcome.    C. Measurement bias:  moderate risk  Reason: intubation adequately described, laparoscopy not adequately described. | | D. Detection bias:  low risk  Reason: outcomes are clearly defined, notably only mortality for laparoscopy deducible from the data  E. Confounding:  high risk  Reason: confounders not sufficiently described, outcomes not specified per confounder.  F. Statistical analysis:  low risk  Reason: analyses adequately performed. | |

Suppl. Table 4

| **Are infants at increased risk of**  **critical events during elective intubation or laparoscopy?** | | | | | |
| --- | --- | --- | --- | --- | --- |
| **Fraser, J. A. *et al.*** Umbilical access in laparoscopic surgery in infants less than 3 months: A single institution retrospective review. *Journal of Pediatric Surgery* **57**, 277-281 (2022)^4^ | | | | | |
| **Study design**  **Treatment era**  **Years of follow-up** | | **Participants** **laparoscopy and intubation** | | **Treatment** | |
| Study design:  Retrospective review  Study era:  2016-2019  Follow-up:  NR  Other information:  operative reports, anesthesia records, postoperative documentation, and postoperative telephone calls to the outpatient surgery clinic were reviewed for complications related to umbilical access. Complications included: bowel or bladder injury upon entry, bleeding umbilical vessels, umbilical vein cannulation, CO 2 embolism, umbilical surgical site infection (SSI), umbilical hernia requiring repair, failed entry requiring conversion to an open procedure, death.  Suppl: Those who did not have operative records available for review were not included in the analysis. | | Type and number of non-participants:  NR, all >3 months were excluded, no other exclusion criteria mentioned.  Type and number of participants:  Total n=365;  Infants <3 months underwent laparoscopic pyloromyotomy (n=246) or inguinal repair (n=119)  Diagnoses/type intervention:  Laparoscopic pyloromyotomy or inguinal repair  Age at intervention:  <3 months, median age 5.9 weeks [IQR: 4.3-8.8]  Median weight 3.9 kg [IQR: 3.4-4.6].  Controls:  NA  Additional study characteristics/ confounders:  No information available about comorbidities. | | Surgery:  laparoscopic pyloromyotomy or inguinal repair  Median operative time: 20 min [IQR: 15-28]; laparoscopic pyloromyotomy: 17 min [IQR: 13-21], laparoscopic inguinal hernia repair: 29 min [IQR: 21-39].  Uniform use of open umbilical entry.  Airway management:  NR  Anesthesia:  General anesthesia  Other:  Intraoperative monitoring by: standard continuous electrocardiogram, pulse oximetry, and end-tidal CO 2 monitoring | |
| **Complications mortality (1w), resuscitation (24h), critical events (24h)** | | | | | |
| **Main outcomes**  **Intubation** | **Main outcomes**  **Laparoscopy** | | **Additional remarks** | |  |
| **NR**  Outcome definitions:  -  Results:  -  Risk factors/ determinants:  - | **Mortality**  Outcome definitions:  Death  Results:  No intraoperative or postoperative mortalities related to the surgical procedure.  Risk factors/ determinants:  NR  **Resuscitation**: NR  **Critical** **events**:  Results:  9 complications (2.5%): 2 cases (pyoloromyotomy) of hypotension and bradycardia upon insufflation (consistent with a response to insufflation) that resolved with desufflation (0.5%) without requiring conversion to open surgery. No signs of CO 2 embolism.  5 superficial umbilical SSIs (1.4%), 1 bowel injury (inguinal hernia repair) upon entry requiring laparoscopic repair (0.2%), 1 umbilical hernia requiring repair 22 days after surgery (0.2%).  No readmission to the hospital or long- term sequela. No complications requiring termination of the procedure or conversion to open, with no intraoperative or postoperative evidence of CO 2 embolism.  Risk factors/ determinants:  All complications were related to intraoperative injury, no reports of intraoperative instability concerning for CO 2 embolism. | | Strengths:  Procedures were consistent with length for OTC  Large sample of infants  Elective surgeries with low risk of complications  Limitations:  No intubation specifics reported.  Retrospective study  No comorbidities mentioned  **Risk of bias**  A. Selection bias:  low risk  Reason: patients <3 months in period were included, no exclusion criteria mentioned. All included is implied.  B. Attrition bias:  unclear  Reason: retrospective cohort, follow-up time not reported    C. Measurement bias:  low risk/unclear  Reason: Laparoscopy low risk: adequately described, intubation method not described: unclear. | | D. Detection bias:  low risk  Reason: outcomes adequately described  E. Confounding:  unclear  Reason: no confounders mentioned, no other comorbidities described.  F. Statistical analysis:  low risk  Reason:  Only descriptive statistics which are performed well, so low risk of bias. |

Suppl. Table 5

| **Are infants at increased risk of**  **critical events during elective intubation or laparoscopy?** | | | | | |  |
| --- | --- | --- | --- | --- | --- | --- |
| **Kalfa, N. *et al.*** Multicentric assessment of the safety of neonatal videosurgery. *Surg Endosc* **21**, 303-308 (2007). ^5^ | | | | | |  |
| **Study design**  **Treatment era**  **Years of follow-up** | | **Participants Intubation** | | **Treatment** | |  |
| Study design:  Retrospective cohort study 7 university hospitals Europe  Study era:  1993-2005  Follow-up:  NR | | Type and number of non-participants:  27 excluded, these were video assisted open surgeries  Type and number of participants:  218 infants younger than 28 days with thoracoscopy or laparoscopy  Diagnoses/type intervention:  Thoracoscopy (n=14)/ laparoscopy (n=204)  Age at intervention:  Mean 16 days (range: 0-28 days)  Mean 3386gr (range: 2,200 - 5,896 g)  Controls:  -  Additional study characteristics/ confounders:  - | | Surgery: different indications gastrointestinal, thoracic, or genital pathologies  Hypertrophic pyloric stenosis: 85  Ovarian cysts: 63  Volvulus with malrotation: 10  Congenital diaphragmatic hernias: 9  Intestinal duplications: 9  Duodenal atresias: 7  Gastro-esophageal reflux: 2  Biliary atresia: 1  Exploratory laparoscopies: liver and extra-hepatic biliary duct examinations: 10  Exploratory laparoscopy complex recto-urinary malformation: 1  Others: 10 (incl: gastric volvulus, gastrostomy, and appendectomy for total aganglionosis)  Operative time: mean 60 min (in 2002) vs 47 min  Airway management: NR  Anesthesia: NR  Other:  Trocar insertion: open technique. 3.5- to 5-mm scope, 2-4 operative trocars were necessary. Ports were 3.5 mm for 2.7-mm instruments.  Mean insufflation pressure: 5.5 mmHg (range, 0–13 mmHg, abdominal suspension n=7) | |  |
| **Complications mortality (1w), resuscitation (24h), critical events (24h)** | | | | | | |
| **Main outcomes**  **Intubation** | **Main outcomes**  **Laparoscopy** | | **Additional remarks** | |  | |
| Outcome definitions:  Desaturation: < 80% with 100% oxygen ventilation  Transient hypotension: requiring vascular expansion  Hypercapnia: >45 mmHg  Hypothermia <34.9 celsius  Results: 26 complications related to poor tolerance of pneumoperitoneum/thorax. 20 during abdominal, 6 during thoracic insufflation.  Desaturation n=8; (Hypoxia was observed more frequently with thoracoscopy) hypotension n=7; hypercapnia n=5; hypothermia n=4 (hypothermia was well tolerated (n=1 bradycardia)); metabolic acidosis n=2; Insufflation temporarily stopped n=9; Insufflation permanently stopped and conversion n=6  Risk factors/ determinants:  Young age, low body temperature, thoracic insufflation, high insufflation pressure and flow, length of surgery. (all p< 0.05)  Pressure of 8 mmHg appeared to be the limit. | **Mortality**  Outcome definitions:  NR  Results:  No mortality occurred  Risk factors/ determinants:  NA  **Other complications**  Outcome definitions:  NR  Results:  16 complications of which 13 deemed minor. 3 redo surgeries  Risk factors/ determinants:  Learning curve of surgeon  Part of the complications are found post-operatively, and part occurred during surgery and were immediately solved. | | Strengths:  Large cohort of infants  Laparoscopic procedures and anesthesia adequately described  Limitations:  Confounders not described  **Risk of bias**  A. Selection bias:  moderate risk  Reason: clearly reported why 27 patients were excluded  B. Attrition bias:  low risk  Reason: there is no follow up. Only problems during surgery are reported    C. Measurement bias:  moderate risk  Reason: reported results are dependent on accurate registration during surgery since the study is retrospective and multi center | | D. Detection bias:  low risk  Reason: outcomes adequately described  E. Confounding:  unclear  Reason: no confounders mentioned, no other comorbidities described.  F. Statistical analysis:  low risk  Reason: Observational data | |

Suppl. Table 6

| **Are infants at increased risk of**  **critical events during elective intubation or laparoscopy?** | | | | | |
| --- | --- | --- | --- | --- | --- |
| **Landisch, R. M., Colwell, R. C. & Densmore, J. C.** Infant gastrostomy outcomes: The cost of complications. *J Pediatr Surg* **51**, 1976-1982 (2016).^6^ | | | | | |
| **Study design**  **Treatment era**  **Years of follow-up** | | **Participants** **laparoscopy** | | **Treatment** | |
| Study design:  Single center Case–Control Study/ Retrospective Comparative Study – Level III  Study era:  January 2011- June 2015  Follow-up:  All 3 month visit.  Other information:  Patients selected via CPT codes 43,246 (PEG) and 43,653 (LG) billing system. Only primary PEGs using the pull technique and reverse transillumination were included. “Revisions of existing gastrostomies, laparoscopic-assisted PEGs, placement by other departments without surgery team involvement, and patients with inadequate records were excluded. Patients who underwent more than one procedure  were included only if operative components could be accounted for by the anesthesia record.”  Outcomes recorded:  mortality, gastrocolic fistula, reoperation (IR or surgical), cellulitis, granulation, wound infections, pneumonia, and tube dislodgement (<6 weeks: 7.6% early tube dislodgements in LG (0 vs. 7.6%, p = 0.01)), bleeding, gastrocolic fistula (GCF), postoperative respiratory failure (defined as requirement of mechanical ventilator support or a significant escalation of support if preoperatively ventilated). | | Type and number of non-participants:  78 PEG  Type and number of participants:  105 laparoscopic gastrostomies (LG)  59.1% male  Mean weight: 4.39 (SD: 1.28) kg  Diagnoses/type intervention:  Laparoscopic gastrostomy  Age at intervention:  All <1y  Mean (SD): 96.3 (70.5) weeks  Controls:  NA  Additional study characteristics/ confounders:  LG infants: significantly younger, higher ASA class, increased frequency of cardiopulmonary disease.  LG gestational age: mean (SD) 35.7 (4.9) weeks  ASA: mean 3.3 (SD: 0.48)  History of: (not further specified)  Neurologic deficit 21.0 %  Pulmonary compromise 36.2 %  Cardiac diagnosis 70.0%  Renal insufficiency 5.7 %  Ventriculoperitoneal shunt 1.9 %  Peritoneal dialysis catheter 1% | | Surgery:  Laparoscopic gastrostomy (indication by individual surgeon)  Abdomen is insufflated, 5-mm telescope through umbilical port. second 5-mm port is positioned under visualization in left subcostal region.  Airway management:  NR  Anesthesia:  General anesthesia  Other:  All received cefazolin preoperatively. 10 surgeons  OR time: mean 130 (SD: 46.4) min | |
| **Complications mortality (1w), resuscitation (24h), critical events (24h)** | | | | | |
| **Main outcomes**  **Intubation** | **Main outcomes**  **Laparoscopy** | | **Additional remarks** | |  |
| **NR**  Outcome definitions:  -  Results:  -  Risk factors/ determinants:  - | Outcome definitions:  Mortality and other perioperative complications  Results:  **Mortality** = 0  Pneumonia (7/105) and post-operative respiratory failure (11/105) in LG group (6.5% vs 10.5%) (n=NR: reported n is calculated from percentage)  Risk factors/ determinants:  NR | | Strengths:  Adequate group of laparoscopic procedures  Limitations:  Perioperative complications not adequately described  **Risk of bias**  A. Selection bias:  low risk  Reason: selection criteria well described  B. Attrition bias:  low risk  Reason: adequate follow up of 3 mo. Notably, loss to follow-up not described.  C. Measurement bias:  moderate risk  Reason: airway not described. Adequate description of laparoscopic procedure. | | D. Detection bias:  moderate risk  Reason: perioperative complications (desaturation etc) not adequately described, other complications, however, are adequately described.  E. Confounding:  low risk  Reason: ASA and other confounders are reported, results not split per risk group  F. Statistical analysis:  low risk  Reason: adequately performed |

Suppl Table 7

| **Are infants at increased risk of**  **critical events during elective intubation or laparoscopy?** | | | | | |
| --- | --- | --- | --- | --- | --- |
| **Meng-Meng, T., Xue-Jun, X. & Xiao-Hong, B.** Clinical effects of warmed humidified carbon dioxide insufflation in infants undergoing major laparoscopic surgery. *Medicine (Baltimore)* **98**, e16151 (2019). ^7^ | | | | | |
| **Study design**  **Treatment era**  **Years of follow-up** | | **Participants** **laparoscopy** | | **Treatment** | |
| Study design:  Randomized intervention study  Study era:  January 2015 to December 2017 Ningbo Women and Children’s Hospital  Follow-up:  NA  Other information:  Inclusion criteria: age <1 year, American Society of Anesthesiology class I or II, use of general anesthesia, normal preoperative body temperature on arrival to the operating room, and surgery time of >2hours.  Exclusion: conversion to open surgery, performance of redo or emergency surgery, and presence of congenital heart or neurological diseases  Transport to the operating room: prewarmed bed wrapped in thermal blanket. operating room temperature: 26°C to 27°C relative humidity: controlled at 30% to 60%. Prewarmed intravenous fluids (at 37°C), preparatory disinfectant at 37°C. During surgery: thermal blanket used:  Group A: standard CO2 at room temperature.  Group B: CO2, warmed and humidified by a photoelectric heating device attached to the insufflation equipment. | | Type and number of non-participants:  NR  Type and number of participants:  63: 38 male, 25 female  Diagnoses/type intervention:  Congenital megacolon, congenital diaphragmatic hernia, and intestinal malrotation.  Age at intervention:  Group A (n=30): 48.12 (8.32) days  Group B (n=33): 51.27 (9.35) days  Controls:  NA  Additional study characteristics/ confounders:  Congenital heart or neurological diseases excluded | | Surgery:  Surgery for congenital megacolon, congenital diaphragmatic hernia, and intestinal malrotation.  Airway management:  NR  Anesthesia:  General: combined intravenous and inhalation anesthesia.  anesthetic regime (determined by anesthesiologist) included: intravenous: atropine, propofol, morphine, fentanyl. Inhaled gases: sevoflurane, desflurane.  Other:  ASA I and II  Operative time, min A: 151.21±31.18 B: 155.16±29.2 p=0.162  Postoperative bowel movement, h A: 55.18±5.92 B: 45.30±4.77 p=0.044  Hospital stay, days A: 13.15±3.76 B: 10.11±2.97 p=0.038 | |
| **Complications mortality (1w), resuscitation (24h), critical events (24h)** | | | | | |
| **Main outcomes**  **Intubation** | **Main outcomes**  **Laparoscopy** | | **Additional remarks** | |  |
| **NR**  Outcome definitions:  -  Results:  -  Risk factors/ determinants:  - | **Mortality**:  Outcome definitions:  NR  Results:  No deaths were noted.  Risk factors/ determinants:  NA  **Resuscitation**: NR  Hypothermia: core body temperature (CBT) < 36°C. measured using: esophageal or rectal probes depending on surgery or anesthesiologist’s preference and access.  **Other** **outcomes**:  cases of reduction in oxygen saturation (SO2) <90%, intraoperative blood loss, postoperative shivering,  Results:  SO2 <90%, n (%) A:2 (6.67%) B:4 (12.12%) p=0.59  Intraoperative blood loss, mL A: 24.32±9.83 B: 22.42±7.51 p=0.541  Postoperative shivering, n (%) A: 4 (13.33%) B: 1 (3.03%) p=0.02  Postoperative hypothermia, n (%) A: 3 (10.00%) B: 1 (3.03%) p=0.032  Risk factors/ determinants:  - | | Strengths:  Randomized cohort  Limitations:  Small sample size  >2h of surgery is not generalizable to the OTC population.  **Risk of bias**  A. Selection bias:  low risk  Reason: inclusion and exclusion criteria adequately described, randomization not described.  B. Attrition bias:  NA  Reason: no follow-up, intervention study    C. Measurement bias:  moderate risk  Reason: anesthesia adequately described, airway not described, laparoscopy not described, intervention adequately described | | D. Detection bias:  low risk  Reason: all outcomes adequately described  E. Confounding:  low risk  Reason: high risk patients excluded and ASA status described.  F. Statistical analysis:  low risk  Reason: well described. |

Suppl table 8

| **Are infants at increased risk of**  **critical events during elective intubation or laparoscopy?** | | | | | |
| --- | --- | --- | --- | --- | --- |
| ***Chinwendu Onwubiko,*** Primary laparoscopic gastrojejunostomy tubes as a feeding modality in the pediatric population ^8^ | | | | | |
| **Study design**  **Treatment era**  **Years of follow-up** | | **Participants** **laparoscopy** | | **Treatment** | |
| Study design:  Single-institution, retrospective review  Study era:  June 2011 and December 2014  Follow-up:  342 days (interquartile range [IQR]=  141–561 days). 53 had >1 year follow-up.  37 died or lost to follow-up at 1 year  Other information:  Inclusion criteria: all patients undergoing primary surgical placement of a GJ tube. Patients with existing GT exchanged for a GJ excluded. | | Type and number of non-participants:  NR  Type and number of participants:  90  47 (52.2%) males  Weight: 5.2 kg (IQR = 4–8.4 kg)  Diagnoses/type intervention:  Indication: gastroesophageal reflux (n = 85, 94.4%), aspiration (n = 40, 44.4%), and failure to thrive (n = 17, 18.9%) (some had multiple indications)  Age at intervention:  5 months (IQR=3–11months) (3weeks-25 years)  Controls:  -  Additional study characteristics/ confounders:  Complex cardiac diseases (n = 34, 37.8%) and respiratory (n = 29, 32.2%) diseases, neurologic disorders (n= 19, 21.1%) not further specified | | Surgery:  primary laparoscopic gastrojejunal (GJ) tube placement. Reference to previously reported method.  Airway management:  NR  Anesthesia:  NR  Other:  - | |
| **Complications mortality (1w), resuscitation (24h), critical events (24h)** | | | | | |
| **Main outcomes**  **Intubation** | **Main outcomes**  **Laparoscopy** | | **Additional remarks** | |  |
| **NR**  Outcome definitions:  -  Results:  -  Risk factors/ determinants:  - | Outcome definitions:  gastric feeding tolerance, subsequent fundoplication, complications, mortality.  Results:  Procedure related **mortality** n=0  Other 30 day mortality: 4.4% (n = 4), total mortality n=21 (23.3%)  GJ-related complications including occlusion, tube migration, and one case of jejunal perforation.  1y gastric feeding tolerance was 34/53 (64.2%)  Risk factors/ determinants:  -  Resuscitation NR  Critical events NR | | Strengths:  First report about this issue.  Part high risk category: mortality within 1 year of procedure = 23%  Limitations:  Retrospective nature, loss of patients  to follow-up given our status as a quaternary referral facility, and by some institution-specific algorithms that limit our practice to be compared with other treatment strategies  **Risk of bias**  A. Selection bias:  moderate risk  Reason: All patients included. However, large group with comorbidities. This may overestimate risk of adverse effects.  B. Attrition bias:  moderate risk  Reason: 53 patients have data of >1 year follow-up. 37 were lost to FU or died. No description of these patients. (Also not number of deceased patients. Notably, since 75% has FU time of 141 days, for our outcome less relevant.)  C. Measurement bias:  moderate risk  Reason: surgical technique is based on previously published techniques. Not further specified. Intubation not specified. | | D. Detection bias:  moderate risk  Reason: outcome mortality specified, other complications only procedure related including occlusion, tube migration, and one case of jejunal perforation, but not further specified.  E. Confounding:  high risk  Reason: cohort includes high risk patients but analyses are not corrected for this.  F. Statistical analysis:  low risk  Reason: descriptive statistic sufficient for our outcome. |

Suppl table 9

| **Are infants at increased risk of**  **critical events during elective intubation or laparoscopy?** | | | | | |
| --- | --- | --- | --- | --- | --- |
| **Ponsky, T. A. & Rothenberg, S. S.** Minimally invasive surgery in infants less than 5 kg: experience of 649 cases. *Surg Endosc* **22**, 2214-2219 (2008) ^9^ | | | | | |
| **Study design**  **Treatment era**  **Years of follow-up** | | **Participants** **laparoscopy** | | **Treatment** | |
| Study design:  retrospective database review USA  Study era:  September 1993 to September 2007.  Follow-up:  NR  Other information:  No exclusions, in all hemodynamically stable on conventional ventilation, transferable to the OR from the NICU were attempted using MIS  Throacoscopic procedures (n=86) (OR time, when reported):  PDA ligation 26 (31 min), TEF 22 (83 min), Lung biopsy 11, Pulmonary lobectomy 10, Lung reduction 2 (66.8 min), Aortopexy 3, Esophageal atresia 3, Pleurodesis 3, Thoracic duct ligation 3, Diagnostic thoracoscopy 1, Division of aortic arch for vascular ring 1, Mediastinal mass excision 1  Complications after thoracoscopic procedures (n=NR):  postoperative allergic arrest after lung biopsy, postoperative respiratory failure after aortopexy in patient with severe tracheomalacia, postoperative pneumothorax following lung biopsy requiring drainage, nonfunctioning vocal cord and aspiration after tracheoesophageal repair, two leaks after TEF repair (treated conservatively with resolution), and gastric perforation after TEF repair from nasogastric tube (NG) placement (n=7, concluded from listed complications) | | Type and number of non-participants:  0  Type and number of participants:  649 (636? 13 missing in table 1)  Diagnoses/type intervention: 43 diagnoses/interventions included (n=550) (86 thoracoscopic excluded, )   - Nissen fundoplication/HH repair ± gastrostomy 310, Redo Nissen 6 - Pyloromyotomy 104, Pyloroplasty 1, Redo pyloromyotomy 1 - Duodenoduodenostomy for DA and AP 20, Pull-through for Hirschsprung’s disease 18, Pull-through for imperforate anus 10, Small bowel atresia 2, Bowel biopsy 1, Bowel resection for stricture 1, Colectomy for NEC 1, Appendectomy 2, Ladd’s procedure 13, - Gastrostomy 10, Gastric duplication resection 1, Repair of gastric perforation 1 - Excision of abdominal mass (teratoma, retroperitoneal mass) 4, Nephrectomy 1, Splenectomy 1 - CDH repair 11 Diaphragm plication 2 - Liver biopsy 5, Cholecystectomy 1 - Ovarian cystectomy 10, Oophorectomy 3, Ovarian biopsy 1 - Inguinal hernia repair 2, Excision of urachal cyst 1 - Lysis of adhesions 5, Diagnostic laparoscopy 1   Age at intervention:  1 day to 14 months (average 8.54 weeks)  weight ranged from 1 to 5 kg (average 3.45 kg).  Controls:  NA  Additional study characteristics/ confounders:  NR | | Surgery:  Laparoscopic  Airway management:  NR  Anesthesia:  NR  Other:  Laparoscopic: 1993: standard 5-mm instruments. After 1994: specially designed 2.8- to 3.4-mm instrumentation  Carbon dioxide insufflation for all procedures (pressure: 10–15 mmHg, flow rate: 1–3 l/min). Initially adult insufflator (1–20 l/min flow rate) led to overinsufflation and distension. 1995: neonatal insufflator.  OR time (average, mean/median NR):  Nissen fundoplication (n=310) 43 min, pyloromyotomy (n=104) 12.5 min,  duodenoduodenostomy (n=20) 76 min, colonic pull-through for Hirschsprung’s disease (n=18) 109.6 min, colonic pull-through for imperforate anus (n=10) 103 min, congenital diaphragmatic hernia repair (laparoscopic) (n=10) 62.5 min. | |
| **Complications mortality (1w), resuscitation (24h), critical events (24h)** | | | | | |
| **Main outcomes**  **Intubation** | **Main outcomes**  **Laparoscopy** | | **Additional remarks** | |  |
| **NR**  Outcome definitions:  -  Results:  -  Risk factors/ determinants:  - | **Mortality**:  Outcome definitions:  Peri-operative death  Results:  Mortality rate: 0.15% (1 death from severe pulmonary hypertension after Nissen (child had multiple medical problems and severe pulmonary disease)  **Critical events**:  Outcome definitions:  NR  Results:  intraoperative complication rate: 0.9%.  overall complication rate: 3% (n=20).  Complications relevant to our outcome included (n=NR):  postoperative respiratory failure from tracheomalacia after Nissen, bladder perforation from a trocar reinsertion, trocar site bleed.  Other laparoscopy complications included:  postoperative bleeding after liver biopsy requiring transfusion, postoperative pleural effusion after diaphragmatic plication requiring drainage, development of hiatal hernia after Nissen, gastrostomy dislodgement 4 days after Nissen/gastrostomy placement, two duodenal perforations during pyloromyotomy (repaired laparoscopically), gastric perforation after pyloromyotomy closed primarily, stoma obstruction after colostomy and pull-through for imperforate anus  Risk factors/ determinants:  NR | | Strengths:  Large cohort with laparoscopic procedures  Limitations:  Unclear discrepancy of 13 missing patients/procedures in table 1  **Risk of bias**  A. Selection bias:  low risk  Reason: all included, exclusion criteria adequately described  B. Attrition bias:  moderate risk  Reason: Follow up time not defined or reported. Some complications were reported after 1 week.    C. Measurement bias:  high risk  Reason: Anesthesia, airway and procedures are not adequately described. Some information is given regarding the insufflation and scopes. Outcomes not relatable to different treatment periods. | | D. Detection bias:  moderate risk  Reason: complications registered were not defined. Unclear if pulmonary and cardiac events perioperatively are registered.  E. Confounding:  high risk  Reason: not corrected for confounders, confounders not specified. However, confounders are present since the confounding factors were reported for the death after Nissen.  F. Statistical analysis:  high risk  Reason:  No statistical analysis performed, insufficient descriptive data reported |

Suppl table 10

| **Are infants at increased risk of**  **critical events during elective intubation or laparoscopy?** | | | | | |
| --- | --- | --- | --- | --- | --- |
| **Walsh, C. M., Ng, J. & Saxena, A. K.** Comparative Analysis of Laparoscopic Inguinal Hernia Repair in Neonates and Infants. *Surgical Laparoscopy, Endoscopy and Percutaneous Techniques* **30**, 459-463 (2020) ^10^ | | | | | |
| **Study design**  **Treatment era**  **Years of follow-up** | | **Participants** **laparoscopy** | | **Treatment** | |
| Study design:  Retrospective single surgeon and associated trainees  Study era:  2013-2018 at Chelsea and Westminster Hospital NHS Foundation Trust, London, UK.  Follow-up:  pediatric outpatient clinic at 6 weeks and 6 months postoperatively. “not every patient returned for follow up”  Other information:  Aim: comparing ≤3 months (corrected premature) to > 3 months (term infants) corrected age at the time of surgery.  - evaluate infants undergoing laparoscopic inguinal hernia repair (prematurity, weight, comorbidities, preoperative hemoglobin)  - compare the outcomes.  Outcomes: demographics, prematurity (<37 wk), corrected age and weight at surgery, preoperative Hb level, comorbidities, anesthetic time (time from arrival into the anesthetic room to time out of theater), major perioperative complications, and inguinal hernia recurrence.  80 cases: no perioperative anesthetic complications. | | Type and number of non-participants:  -  Type and number of participants:  N=80 <12mo  67 (84%) male  median weight: 5.5 kg (range 2.1 to 10.8).  <3mo 4.7 (range 2.1 to 7.2)  >3mo 6.7 (range 4.6 to 10.8) kg (P<0.001)  Diagnoses/type intervention:  47 (59%) unilateral inguinal hernia repair (60% right-sided, 40% left-sided)  33 (41%) bilateral repair.  Age at intervention:  median corrected age 10.5 (range -2.5 to 44) weeks  Controls:  -  Additional study characteristics/ confounders:  significant anesthetic comorbidities (chronic lung disease and cardiac defects) were recorded. 59% were premature, 33% weighed < 5 kg at surgery, 16% significant cardiac and/or respiratory comorbidities.  N=12 significant comorbidity  <3mo: 8: 2 cardiac, 7 respiratory;  >3mo: 4: 2 cardiac, 2 respiratory. P=0.532  (atrial and/or ventricular septal defects, chronic lung disease) n=1 (>3mo) previously open duodenal atresia repair (5 mo before LIHR).  preoperative hemoglobin median:  <3mo 105 (range 79 to 200)  >3mo 112 (range 77 to 169) g/L p= 0.051 | | Surgery:  laparoscopic inguinal hernia repair (LIHR) operative technique:  A single 5-mm nonballoon umbilical port was used to induce pneumoperitoneum and admit a 5-mm 30-degree laparoscope. Two 3-mm instruments were directly inserted via lateral stab incisions on the left and right side. herniorrhaphy with 4/0 Prolene purse-string suture umbilical incision was closed with 2/0 Vicryl (Polyglactin 910, Ethicon Inc.) purse-string suture of the fascia and skin glue lateral stab incisions were closed with skin glue only.  Used anesthetic time as surrogate marker for operating time. Median anesthetic time: 81 minutes unilateral laparoscopic inguinal hernia repairs; 94 minutes bilateral laparoscopic inguinal hernia repairs.  Airway management:  NR  Anesthesia:  Median anesthetic time  ≤3mo: 93 min (range 61-125)  >3mo: 83 min (range 47-146) (P= 0.001)  Pain relief: Simple analgesia (paracetamol or ibuprofen) or, if required, opiate analgesia  Other:  Postoperatively: neonatal intensive care unit, neonatal high dependency unit, or pediatric ward for ventilation or apnea monitoring. | |
| **Complications mortality (1w), resuscitation (24h), critical events (24h)** | | | | | |
| **Main outcomes**  **Intubation** | **Main outcomes**  **Laparoscopy** | | **Additional remarks** | |  |
| Outcome definitions:  NR  Results:  No anesthetic complications  Median anesthetic time unilateral LIHR <3 mo: 87 (range 61-125) min, >3 mo: 75 (range 47-146) min  Bilateral <3 mo: 100 (range 69 - 121) min, >3 mo: 81 (range 50 - 125) min.  Risk factors/ determinants:  NR | **Mortality**:  Outcome definitions:  Mortality  Results:  No mortality  Risk factors/ determinants:  N=0  **Resuscitation**:  Outcome definitions:  Perioperative complications, not further specified.  Results:  No perioperative complications  Risk factors/ determinants:  NR | | Strengths:  Comparison made <3 months and above.  Generalizability due to surgeon and the associates, teaching hospital situation  Limitations:  Single surgeon evaluation.  Total active anesthetic time instead of operating time, anesthesia time may be longer in younger patients even if the surgery is not.  Retrospective study, with missing data not consistently documented, including length of stay postoperatively, as many infants on neonatal intensive care unit (NICU) would have remained on NICU postoperatively for other nonsurgical reasons. No data collected on the proportion of patients and indication for remaining intubated and ventilated on NICU postoperatively.  **Risk of bias**  A. Selection bias:  moderate risk  Reason: single surgeon, so other surgeons patients not included. Unclear if other surgeons performed the procedure and how many patients that would be and any characteristics for them.  B. Attrition bias:  moderate risk  Reason: no follow-up time reported. Discussion states not all patients reported for FU check-up, n=NR.  C. Measurement bias:  moderate risk  Reason: laparoscopy adequately described, intubation not described. | | D. Detection bias:  low risk  Reason: complications adequately described  E. Confounding:  low risk  Reason: comorbidities adequately described, descriptive statistics not corrected for confounders.  F. Statistical analysis:  low risk  Reason: statistical analyses performed appropriately. T test and Fisher exact test (P< 0.05 significance) |

**References:**

1 Beltman, L. *et al.* Risk factors for short-term complications graded by Clavien-Dindo after transanal endorectal pull-through in patients with Hirschsprung disease. *J Pediatr Surg* **57**, 1460-1466, doi:10.1016/j.jpedsurg.2021.07.024 (2022).

2 Chou, C. M., Yeh, C. M., Huang, S. Y. & Chen, H. C. Perioperative parameter analysis of neonates and infants receiving laparoscopic surgery. *J Chin Med Assoc* **79**, 559-564, doi:10.1016/j.jcma.2016.05.005 (2016).

3 Disma, N. *et al.* Neonates undergoing pyloric stenosis repair are at increased risk of difficult airway management: secondary analysis of the NEonate and Children audiT of Anaesthesia pRactice IN Europe. *Br J Anaesth* **129**, 734-739, doi:10.1016/j.bja.2022.07.041 (2022).

4 Fraser, J. A. *et al.* Umbilical access in laparoscopic surgery in infants less than 3 months: A single institution retrospective review. *Journal of Pediatric Surgery* **57**, 277-281, doi:doi:10.1016/j.jpedsurg.2021.11.010 (2022).

5 Kalfa, N. *et al.* Multicentric assessment of the safety of neonatal videosurgery. *Surg Endosc* **21**, 303-308, doi:10.1007/s00464-006-0044-1 (2007).

6 Landisch, R. M., Colwell, R. C. & Densmore, J. C. Infant gastrostomy outcomes: The cost of complications. *J Pediatr Surg* **51**, 1976-1982, doi:10.1016/j.jpedsurg.2016.09.025 (2016).

7 Meng-Meng, T., Xue-Jun, X. & Xiao-Hong, B. Clinical effects of warmed humidified carbon dioxide insufflation in infants undergoing major laparoscopic surgery. *Medicine (Baltimore)* **98**, e16151, doi:10.1097/MD.0000000000016151 (2019).

8 Onwubiko, C. *et al.* Primary laparoscopic gastrojejunostomy tubes as a feeding modality in the pediatric population. *J Pediatr Surg* **52**, 1421-1425, doi:10.1016/j.jpedsurg.2017.05.015 (2017).

9 Ponsky, T. A. & Rothenberg, S. S. Minimally invasive surgery in infants less than 5 kg: experience of 649 cases. *Surg Endosc* **22**, 2214-2219, doi:10.1007/s00464-008-0025-7 (2008).

10 Walsh, C. M., Ng, J. & Saxena, A. K. Comparative Analysis of Laparoscopic Inguinal Hernia Repair in Neonates and Infants. *Surgical Laparoscopy, Endoscopy and Percutaneous Techniques* **30**, 459-463 (2020).
